# Endotracheal Tube Fastening Device-Related Facial Pressure Ulcers

**DOI:** 10.7759/cureus.16796

**Published:** 2021-07-31

**Authors:** Vaibhav Rastogi, Abraham J Layon

**Affiliations:** 1 Internal Medicine, Lake City Medical Center, Lake City, USA; 2 Anesthesiology and Critical Care, The University of Central Florida College of Medicine, Orlando, USA

**Keywords:** pressure ulcer, endotracheal tube, intubation, mechanical ventilation, covid19

## Abstract

Acute respiratory distress syndrome (ARDS) can be present in a substantial number of hospitalized Coronavirus disease 2019 (COVID19) disease patients. Some of these patients progress to severe ARDS and require mechanical ventilation. Patients requiring mechanical ventilation and intensive care unit (ICU) admission are at an increased risk of developing pressure ulcers from multiple medical devices used in their care. In this report, we describe a case of facial pressure ulcers in a 59-year-old COVID19 positive female with ARDS requiring endotracheal intubation and mechanical ventilation.

## Introduction

A pressure ulcer, also known as pressure injury, is a confined damage to the skin and soft tissues from the increased mechanical load which could originate from the bodyweight itself or any medical device. Medical device-related pressure ulcers are one of the common complications seen in the intensive care units (ICU). They impede the overall healing of patients as well as increase the financial burden on the already burdened healthcare system [1].

Approximately 16% of Coronavirus disease 2019 (COVID19) pneumonia-induced acute respiratory distress syndrome (ARDS) patients may require endotracheal intubation, with an endotracheal tube (ETT) being adhered to the face with the help of an ETT fastener [2]. Proning has been shown to improve mortality in these patients [3]. The combined effect of bodyweight forces (proning) and medical device-related forces in these patients can increase the risk of the development of facial pressure ulcers. Here, we report a case of pressure ulcers on the face from the commercially available ETT fastener in a COVID19 patient.

## Case presentation

A 59-year-old caucasian woman with a past medical history of chronic obstructive pulmonary disease, morbid obesity presented to the emergency department complaining of fevers, dry cough, and loss of taste and smell for about ten days prior to presentation. The woman tested positive for severe acute respiratory syndrome coronavirus 2 (SARS-CoV-2) approximately nine days before admission.

Upon admission, vital signs included temperature 35.8° C (96.4° F), heart rate 116 beats per minute, respiratory rate of 22 breaths per minute, non-invasive blood pressure 138/67 mm Hg, and SpO2 67% on room air. An arterial blood gas analysis showed pH 7.4, PaCO2 36.9 mm Hg, PaO2 53 mm Hg; pertinent laboratory data upon admission included white blood cells 15,200 cells per microL, blood urea nitrogen 18 gm/dL, creatinine 1.32 gm/dL, aspartate transaminase 48 U/L. Inflammatory markers were: D-dimer 1.58 mg/L, ferritin 465 nanogram/mL, C-reactive protein 34.4 mg/dL, lactate dehydrogenase 619 U/L. Portable chest radiography at the time of admission showed bibasilar airspace disease. Pulmonary embolus protocol- computerized tomographic angiography showed severe, widespread bilateral ground glass (alveolar) airspace disease consistent with COVID19 pneumonia; no pulmonary embolism was noted.

The patient was initially placed on Bimodal Positive Airway Pressure (BiPAP) for hypoxia and was admitted to our step-down unit. She was treated with the then prevailing regimen of anti-viral remdesivir, high-titer anti-SARS-CoV-2 convalescent plasma, dexamethasone, and empiric therapeutic anticoagulation (shared decision making with the patient). Despite therapy and supportive care, she deteriorated and needed to be transferred to the ICU 12 days after admission.

On day #13 of this admission, the patient was endotracheally intubated and the ETT secured with a commercially available fastener {Hollister AnchorFast oral endotracheal tube fastener (Part # 9799), Libertyville, IL}. During the ICU course, she required vasopressors intermittently and daily prone sessions for severe ARDS. As she could not be weaned off from the ventilator, percutaneous tracheostomy (Cook Medical, Blue Rhino percutaneous tracheostomy, Bloomington, IN) was performed on day 21 of intubation.

**Figure 1:**
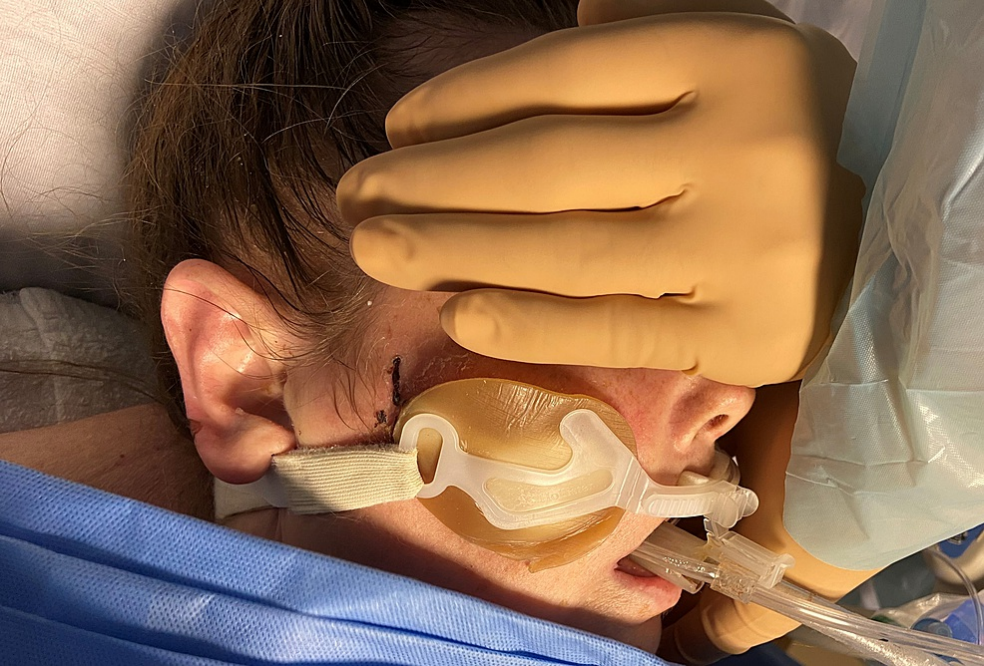
Intubated patient with Endotracheal tube fastener skin barrier pads.

**Figure 2:**
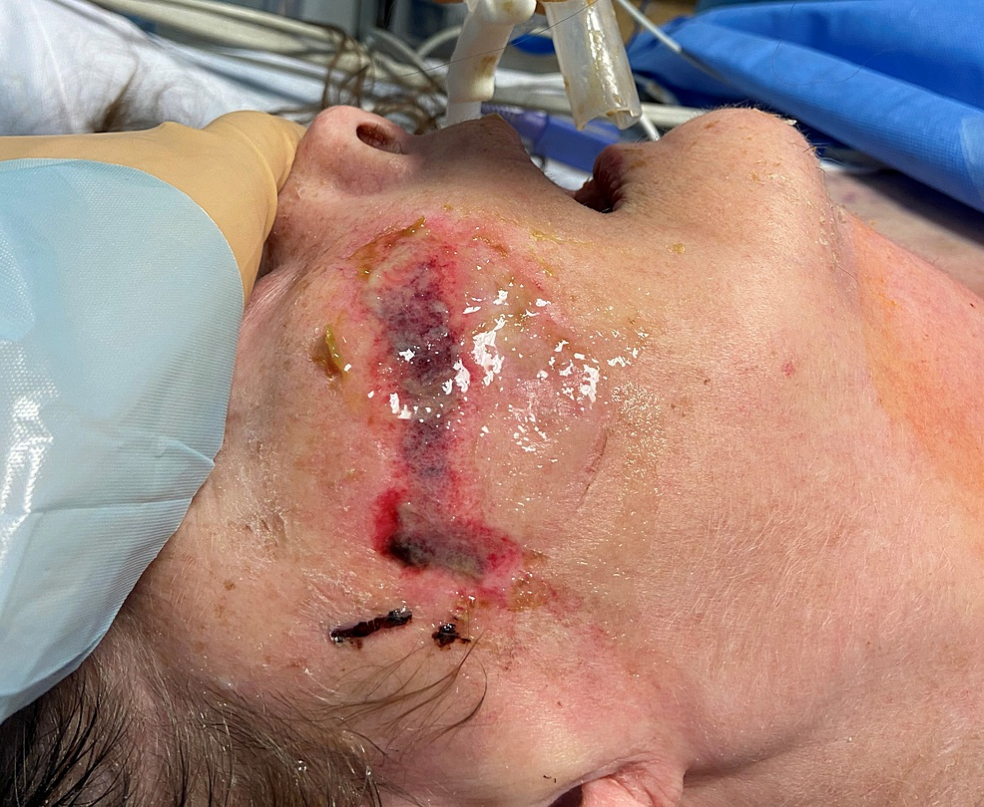
Stage 2 pressure ulcer at the site of endotracheal tube fastener skin barrier pad.

After the tracheostomy had been inserted and was affixed in place, the Hollister AnchorFast oral endotracheal tube holder was gently removed, revealing - under the skin barrier pads - bilateral stage 2 pressure ulcers on her face; these had not been present prior to placement of the affixation device when the patient was endotracheally intubated (Figures 1, 2).

While the pressure ulcers were managed with frequent (two times a day) cleaning with saline and the application of mupirocin our patient’s medical condition continued to deteriorate and, on day #31 post-intubation, her family (surrogate decision-maker) chose to withdraw life-sustaining treatment.

## Discussion

Although preventable, medical device-related ulcers may be noted in up to one-third of the hospitalized patients [4]. In their study of 483 critically ill patients in multiple centers throughout the USA and Australia, Coyer et al noted that the prevalence of device-related pressure ulcers was 3.1% [5]. ETTs and nasogastric tubes were the most often implicated devices for these ulcers. These investigators noted an increased risk of medical device-related ulcers in men, those who are overweight, and patients with a prolonged ICU stay [5]. Although a woman, our patient had a body mass index (BMI) of 42, was being proned, and had a prolonged ICU stay of more than 30 days. These factors may have attributed to the development of facial pressure ulcers at the site of skin barrier pads placement. Proning plays an important role in the development of facial ulcers as indicated by Shearer et al who observed that 47.6% proned COVID-19 patients developed facial pressure ulcers with 84% of them developing ulcers on the cheeks similar to our patient. They also noted a positive association between the risk of facial pressure ulcers and prolonged proning [3].

A perfect ETT securing device is one that can prevent any ETT slippage (displacement) as well as unplanned tracheal extubations, prevent any pressure ulcers in the skin and neck area [6], and is easy to adjust. Unfortunately, no such ideal device presently exists, and all the current ETT securing devices carry their pros and cons [7]. Fisher et al compared the commercial and non-commercial ETT securing devices, observing that commercially available devices exert a higher force on the face, which may increase the risk of pressure ulcers. Conversely, such commercial devices permit rapid and easy movement of the ETT, which may decrease the risk of pressure ulcer from the tube itself [7]. Further, Landsperger et al. noted that ETT fasteners are better than adhesive tape in decreasing the incidence of lip ulcers, facial skin tears, and ETT dislodgement [8].

The risk of pressure ulcers from ETT securing devices increases by a factor of six when vasopressors are used [6]. Thus, patients on vasopressors should have more surveillance of pressure ulcers. Protocol use, such as mandating frequent repositioning of the ETT every four hours and changing the ETT securing device every four days can prevent the development of pressure ulcers but this can be labor-intensive. Frequent repositioning of ETT will also permit more frequent assessments [6]. Coyer et al also observed that frequent repositioning of the tubes prevented the injury [5]. Silicone-based thin foam dressings used as a barrier between the skin and ETT fastener can prevent the development of facial ulcers [3].

## Conclusions

Medical device-related pressure ulcers are commonly seen in hospital settings. In the ICU, ETTs are responsible for the majority of pressure ulcers and ETT fasteners can cause facial pressure ulcers. These can be prevented by frequent repositioning of ETT along with regular changing of the ETT fastener. However, currently, there are no proven strategies to prevent pressure ulcers from ETT fasteners. Properly powered, prospective, and multicentered studies are needed to evaluate strategies to reduce these iatrogenic injuries.
